# Effect of a Single Bout of Acute Aerobic Exercise at Moderate-to-Vigorous Intensities on Motor Learning, Retention and Transfer

**DOI:** 10.3390/sports8020015

**Published:** 2020-01-29

**Authors:** Håvard Lorås, Monika Haga, Hermundur Sigmundsson

**Affiliations:** 1 Department of Teacher Education, Faculty of Social and Educational Sciences, NTNU—Norwegian University of Science and Technology, 7491 Trondheim, Norway; monika.haga@ntnu.no; 2 Department of Psychology, Faculty of Social and Educational Sciences, NTNU—Norwegian University of Science and Technology, 7491 Trondheim, Norway; hermundur.sigmundsson@ntnu.no; 3 Department of Sport Science and Physical Education, Reykjavík University, IS-101 Reykjavik, Iceland

**Keywords:** endurance training, motor skill, practice, arousal

## Abstract

Acute exercise influences human cognition, and evidence suggests that learning can be improved. According to the cognitive–energetic approach towards exercise cognition, exercise represents a stressor that elevates physiological arousal, which, in turn, increases the availability of mental resources. However, the degree of arousal is hypothesized to have optimal and suboptimal states, and moderate intensity exercise is thus considered to be favorable compared to low intensity and vigorous exercise. The current evidence for such a moderating effect of exercise intensity on motor learning, however, appears somewhat mixed. Therefore, the purpose of this study was to explore the effect of aerobic exercise conducted with different exercise intensities on immediate practice, transfer, and 24-h retention of a motor skill. To this end, young adults (n  =  40, mean (SD) age: 23.80 (1.98) years) were randomized to exercise at either 50% or 75% of age-predicted maximal heart rate according to the Karvonen formulae. Immediately after exercising, participants practiced a high-precision golf putting task in a blocked design. Retention and transfer of skill were assessed after 24 h. Results indicated that both groups demonstrated motor learning, retention, and transfer at a similar level. Further works are thus needed to establish the specific relationship between exercise and learning and establish the factors that have an influence.

## 1. Introduction

A single bout of acute cardiovascular exercise performed just before practice is hypothesized to influence learning in a time-dependent fashion by an effect on the processes involved in encoding and consolidation [1]. Meta-analysis of reports where memory tasks constituted the measure of performance has shown a moderate positive effect of acute aerobic exercise on short-term and long-term memory [2] as well as on aspects of working memory processes [3].

Motor learning might be especially prone to exercise-induced improvements, as endurance exercise can facilitate motor learning-related neuroplasticity [4]. Several mechanisms potentially underlying the effect of acute exercise on motor learning have been identified [5]. First, skeletal muscles can act as endocrine organs capable of secreting molecules that increase the availability of neurochemicals relevant for neuroplasticity [6]. Lactate released from working skeletal muscles might thus significantly contribute to brain metabolism and modulate several brain processes [7,8]. In addition, acute exercise may impact neuroplasticity in cortico-spinal pathways [9] as well as increase the excitability of central brain areas involved in motor learning, such as the primary motor cortex and supplemental motor area [10]. These latter mechanisms might positively affect motor memory and thus improve motor retention [11].

In acute aerobic exercise, intensity has long been suggested to be an important parameter that modulates exercise-induced effects on cognition and learning [12]. Intensity is usually expressed as a percentage relative to a maximal power output, oxygen consumption, and/or heart rate [13]. In the cognitive–energetic approach towards exercise cognition [14], exercise is viewed as a stressor that impacts the level of arousal, i.e., the state of being awoken/stimulated [15]. According to this approach, an increase in intensity is expected to introduce a joint increase in the level of arousal, which facilitates the efficiency of cognitive processes by engaging more resources, such as attention and focus, during complex and effortful learning tasks [16,17].

However, the relationship between arousal (or exercise intensity) and task performance is not expected to be linear. One can trace the lineage of this hypothesis back to the Yerkes and Dodson inverted-U theory concerning the effect of arousal on task performance. The law basically dictates that performance increases with elevated arousal up to a certain point. When levels of arousal become too pronounced, task performance is compromised [18]. Cognitive–energetic theories originating from this early work have usually included this as a basis for hypothesizing that moderate intensity exercise equates to moderate levels of arousal, suggesting that optimized performance is expected, while heavy exercise equates to over-arousal, resulting in a performance decline returning to an initial level as that at rest. Thus, cognitive–energetic models suggest that moderate intensity exercise-induced arousal generates a positive effect on learning, while low and high levels of exercise-induced arousal introduces negative effects [19].

A non-linear relationship between exercise intensity/arousal and task performance is also postulated in neuroendocrinological models of exercise cognition [15,20]. During exercise, the sympathoadrenal system is initiated by the hypothalamus and brainstem. This results in the release of catecholamines, and moderate intensity exercise also induces the release of epinephrine and, to a lesser extent, norepinephrine. Heavier exercise, in turn, generates substantial increases in both norepinephrine and epinephrine. There are thus significant increases in the brain concentrations of catecholamines following acute exercise [21]. Moderate intensity exercise further activates several brain areas due to the elevated levels of catecholamines, leading to facilitated sensation and perception by increasing the signal to ‘noise’ ratio. Substantial levels of catecholamines at heavy exercise, however, might disrupt the signal to ‘noise’ ratio, hence inhibiting sensation and perception. This neuroendocrinological theory [15,20] postulates that moderate intensity exercise will facilitate the learning of psychomotor skills, due to moderate increases in the concentrations of brain neurotransmitters [22]. During vigorous exercise, on the other hand, different physiological changes might be engaged and impair the motor learning process [23,24,25].

In the past two decades, evidence for a nonlinear relationship between task performances and levels of exercise-induced arousal has emerged in various fields. In two separate meta-analyses, McMorris and colleagues examined the results from studies investigating the effect of acute exercise at different intensities on the performance of whole-body psychomotor skills [26], and the effect of acute exercise at different intensities on the performance of cognitive tasks, e.g., reaction time, attention, concentration, and decision making [3]. The meta-analytical measures indicated that acute moderate intensity exercise generated a larger effect compared to low and vigorous intensities for cognitive tasks capturing information-processing speed, indicating that increased arousal from moderate intensity exercise results in faster processing speed [3]. However, performance in psychomotor tasks after acute exercise appeared to be poorer after high intensity workouts, while moderate exercise-induced arousal resulted in small and nonsignificant effect sizes. The authors thus suggested that in tasks combining perception and action, the complex relationship introduces different effects of acute exercise on task performance compared to effects on tasks that can be considered to rely more strongly on cognitive processes [26].

In recent work on perceptual motor learning, the results appear to be somewhat mixed. A single bout of aerobic exercise conducted at moderate intensity has been shown to improve motor learning of thumb abduction [27], simulated surgery [28], and isometric pinching [29]. Snow et al. (2016), however, did not report significant effects on motor learning in a tracking task [30]. A similar divergence of results has been reported in studies on the immediate effect of heavy exercise on perceptual motor learning, where an improvement [31], no effect [32], and reduction [10] in motor learning outcomes has been found. By the authors’ knowledge, the only study directly comparing the effect of different exercise intensities upon motor learning was Thomas et al. (2016), in which ther results indicated that heavy intensity introduced better retention in a tracking task compared to very low intensity exercise. In the latter study, however, exercise bouts were conducted after motor practice [33].

Thus, theoretical frameworks postulate positive effects of moderate intensity exercise on motor learning. Moreover, a mixture of findings has emerged in previous works that have investigated the effects of aerobic exercise on motor learning. Based on the presented considerations, the current study set out to further examine if acute aerobic exercise conducted at moderate-to-vigorous intensity leads to different rates of motor acquisition, transfer, and retention. Assessments of working memory and self-reported arousal were also included in order to establish whether the exercise introduced the theoretically expected cognitive effects.

## 2. Materials and Methods

### 2.1. Participants

Following approval from the regional ethics committee for medical research, 40 healthy participants (20 male/20 female) were recruited from a university community. All participants were healthy and without any neurological complaints (see Table 1 for demographics) and completed the Physical Activity Readiness Questionnaire (PAR-Q) before participating in the experiments. The PAR-Q [34] is intended to detect if participants are at risk when increasing their exercise level. Those who answer yes to one or more of seven questions are advised to consult their doctors. In the current study, all participants answered no to all questions. Participants reported no previous specific golf putting experience or practice, besides conducting a few holiday-type miniature golf games. The study protocol was approved by the regional committee for medical and health research ethics (REC Central). All subjects provided informed written consent prior to participating in the study, and all procedures were in accordance with the tenets of the Declaration of Helsinki.

### 2.2. Procedures

**Figure 1:**
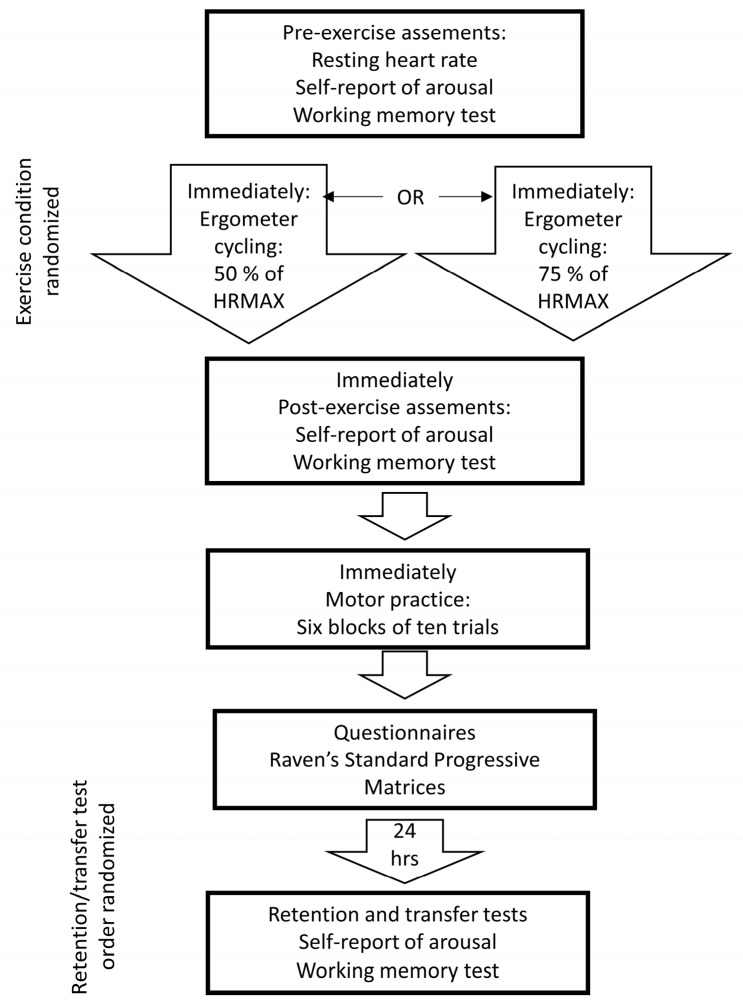
Overview of the experimental procedures.

The total group of participants was divided into two groups by selecting a number generated by Research Randomizer [35]: One group conducting ergometer cycling at moderate intensity and another group cycling at vigorous intensity. Participants completed two visits to the lab (see Figure 1). On day one, they were equipped with heart rate monitors (Polar M400, Polar, Finland) and lay down on a couch for 5 min in order to measure the resting heart rate. Next, they completed a self-report of arousal and the working memory test. These assessments were followed by 25 min of ergometer cycling at the designated exercise intensity. Immediately after exercising, participants reported their arousal and were re-tested on the working memory task. The final steps of the lab visit consisted of practice and testing on the golf putting task and completing the physical activity and sedentary time questionnaires as well as the Raven’s Standard Progressive Matrices (R-SPM, see description below). Twenty-four hours after their first visit, participants returned and completed the self-report of arousal, working memory test, and retention/transfer testing on the golf putting task.

### 2.3. Questionnaire

Demographical data and information on the levels of physical activity were obtained with the International Physical Activity Questionnaire (IPAQ) [36,37]. Participants further reported background information on sedentary behavior via the Sedentary Behavior Questionnaire (SBQ), which consisted of nine items on sedentary behavior on weekdays or weekend days [38].

### 2.4. Cognitive Assessments

The standard version of the Raven’s Standard Progressive Matrices (R-SPM) was used to assess non-verbal ability [39]. This test mainly measures psychometric general intelligence [40]. Participants were presented with 60 pattern-matching matrices, in which one small section was removed, and chose the one they perceived to fit the missing section. There was no time limit and the raw score was used for further analysis. 

Working memory was assessed by the Letter-Number-Sequencing subtask from the Wechsler Adult Intelligence Scale [41]. In this task, participants listened to numbers and letters (e.g., 5-J-3-C-8, K-9-B-5-D) and then first repeated back the numbers in ascending order, followed by the letters in alphabetical order [42]. The raw score for the maximal n of correct numbers and letters repeated was used for further analysis.

### 2.5. Self-Reported Arousal

Perceived arousal was measured by a 6-item Perceived Arousal Scale (PAS), which consisted of 6 adjectives rated on a 7-point Likert scale (1  =  not at all to 7  =  extremely). Three of the adjectives reflected high arousal (e.g., energetic), whereas the other 3 reflected low (or lack of) arousal [43,44]. A total arousal score was computed by reverse scoring the low-arousal subscale and then aggregating the items. Thus, a higher total score indicates higher arousal. Cronbach’s alpha was 0.85 for the total arousal score.

### 2.6. Aerobic Exercise

The participants pedaled an individually adjusted (seat height) ergometer (Sportsmaster Studio U400, Nordic Sportsmaster, Nesbru, Norway) for 25 min, including a 5-min warm-up at 100 watts. The target heart rate for exercise intensity was determined by the Karvonen formulae [45]: Target Heart Rate = ((HR_MAX_ − HR_REST_) × % target intensity (50% or 75%)) + HR_REST_. HR_MAX_ was estimated by applying the formulae 211 - 0.64 × age, which has shown an excellent fit for people in their twenties [46]. HR_REST_ was measured from the last minute of lying down [47]. Intensity was continuously monitored by the heart rate monitors.

### 2.7. Golf Putting Task

The learning task consisted of participants putting standard golf balls (Warbird Plus, Callaway, Carlsbad, California, US) to a circular target 3 m from the starting point on an artificial indoor putting green (size: 1.5 m × 3.5 m, 16 mm putting green turf) using a standard putter (Cleveland Putter Huntington Beach, Cleveland Golf, Huntington Beach, California, US). The target, marked in red, equaled the size of regular golf balls (43 mm in diameter). Participants were asked to accurately putt the golf ball to the target, on which the ball was supposed to stop.

Before entering the lab experiment, participants watched a short video of putting performed by a skilled golfer [48]. Before conducting their first putt, they were briefly presented with five basic concepts [49] of the golf putt (gripping, how to stand, pointing club, moving with shoulders, and hitting ‘through’ the ball). Each participant thereafter performed two warm-up putts and practiced six blocks of 10 putts [50,51]. Importantly, no specific feedback other than the visible results of the putt (i.e., knowledge of the result) was given to the participants. That is, no additional information, such as technical feedback (i.e., knowledge of performance), was provided during the practice [49]. Twenty-four hours after practicing, participants performed two warm-up putts and one block of 10 putts as a retention test, as well as one block of 10 putts standing 0.5 m closer to the target as a transfer task.

Putting performance was measured with a video camera (Panasonic digital camcorder, Osaka, Japan) positioned right next to the putting green perpendicular to the target. The camera was placed on a level tripod to allow a good capture of the final golf ball position after each putt. The recordings were obtained with a resolution of 30 Hz. The focus and aperture were adjusted until the camera produced clear images. Recordings were analyzed offline by means of the open-source software Kinovea [52], which was used to analyze all images and videos from the camera. All the recorded video was first checked for any obvious errors. Kinovea was thereafter used to locate the target position (reference point) as well as the final golf ball position for each of the 3200 trials. The shortest distance from the ball to the target (absolute radial error) in centimeters was used as measure of performance [53].

### 2.8. Statistical Analysis

Histograms, Q-Q plots, and Kolmogorov–Smirnov tests were used to establish normality assumptions. Absolute error, averaged across blocks of 10 trials, was examined with a 2 (group: moderate intensity versus vigorous intensity) × 6 (blocks of 10 trials) repeated-measures analysis of variance (ANOVA), with repeated measures on the last factor for the practice phase. The *p*-value was Bonferroni corrected in post hoc comparisons, with partial eta squared (*ղ2p*) as the indicator of the effect size (interpreted [54] as low <0.04, medium ≥0.04 to <0.36, and large ≥0.36). Independent samples *t*-tests were utilized for the retention and transfer tests, with Cohens *d* as a measure of the effect size, in which 0.2, 0.5, and 0.8 were considered small, moderate, and large, respectively [54]. As an initial analysis, we first established that there was no significant difference between males and females in motor learning (*F* (2, 38) = 0.17, *p* = 0.95, *ղ2p* = 0.005, small). Predictive Analytics Software (PASW, IBM, US; previously SPSS) Version 25.0.0 was applied for statistical calculations, with *p* < 0.05 as the statistical significance criterion.

## 3. Results

Descriptive information regarding the study groups is provided in Table 1, which indicates that none of the demographical variables were significantly different between groups.

### 3.1. Motor practice

**Figure 2:**
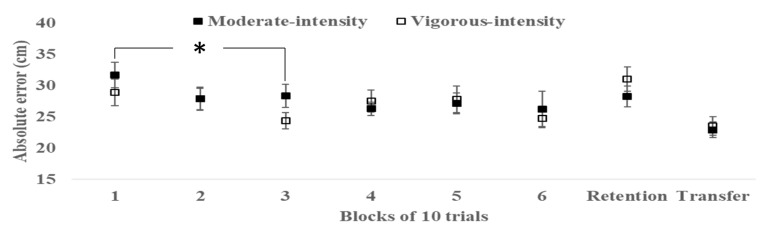
Putting accuracy (lower scores indicate greater accuracy) of the moderate intensity and vigorous intensity groups during motor practice, 24-h retention, and transfer. *Error bars* indicate standard errors. * Main effect of block (*p* < 0.01).

As depicted in Figure 2 (left-hand side), there was no significant difference (*t* (1, 38) = 0.95, *p* = 0.35, *d* = 0.31) between groups in putting accuracy in the first practice block (moderate intensity: mean (SD) 31.68 (9.40), vigorous intensity: mean (SD) 28.90 (9.04), mean difference 2.78 95% CI (low, high): −3.16, 8.73), which suggests similar initial performance levels. Additionally, as clearly visible in Figure 2, both groups increased their putting accuracy across practice blocks. Thus, the main effect of block was significant (*F* (5, 38) = 5.23, *p* = 0.03, *ղ2p* = 0.12, medium). Post hoc analysis indicated that this main effect originated from an improvement from block#1 towards block #3. However, no significant group x block interaction was observed (*F* (5, 38) = 0.39, *p* = 0.53, *ղ2p* = 0.01, small).

### 3.2. Motor Transfer and Retention

No significant difference was found between the moderate intensity group and the vigorous intensity group on either retention (*t* (1, 38) = 1.10, *p* = 0.28, *d* = 0.36, small) or transfer (*t* (1, 38) = 0.33, *p* = 0.75, *d* = 0.11, small) of putting accuracy (see Figure 2, right-hand side).

### 3.3. Working Memory

**Figure 3:**
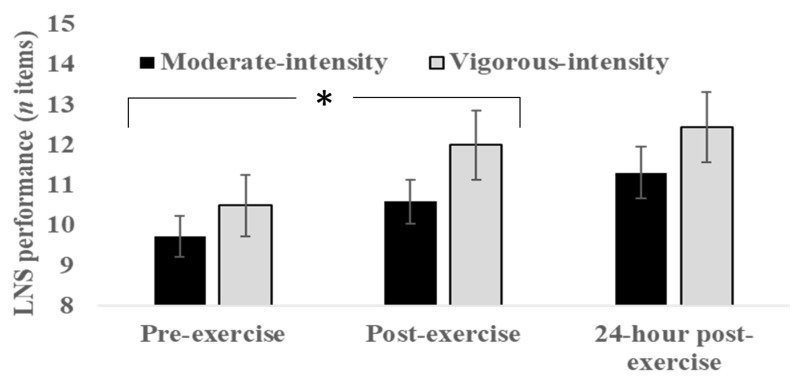
Working memory performance of the moderate intensity and vigorous intensity groups before and immediately after exercise, and at 24-h post exercise. *Error bars* indicate standard errors. * Main effect of exercise (*p* < 0.01).

As can be seen in Figure 3, both groups showed improved working memory performance as captured by the LNS test from pre- to post exercise. Repeated-measures ANOVA indicated an overall significant effect of timepoint on working memory performance (*F* (2, 38) = 15.30, *p* = 0.000, *ղ2p* = 0.29, large). Post hoc pairwise comparisons suggested that this was due to a significant difference between pre-exercise and post-exercise assessments (*p* < 0.001). No significant group x time interaction was found (*F* (2, 38) = 1.01, *p* = 0.48, *ղ2p* = 0.01, small). The moderate intensity group demonstrated a large effect of exercise on working memory (*d* = 0.84, large) and the vigorous intensity group a moderate effect (*d* = 0.84, moderate).

### 3.4. Self-Reported Arousal

**Figure 4:**
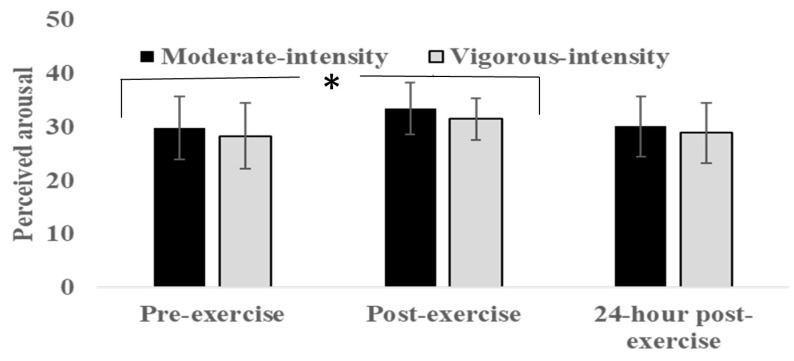
Self-perceived arousal of the moderate intensity and vigorous intensity groups before and immediately after exercise, and at 24-h post-exercise. *Error bars* indicate the standard deviation. * Main effect of exercise (*p* < 0.01).

The mean (SD) of the self-perceived arousal across study groups can be found in Figure 4. Repeated-measures ANOVA indicated an overall significant effect of timepoint on the perceived arousal (*F* (2, 38) = 5.84, *p* = 0.004, *ղ2p* = 0.13, medium). Post hoc pairwise comparisons suggested that this was due to a significant difference between pre-exercise and immediate post-exercise assessments (*p* < 0.001). No significant group x time interaction was found (*F* (2, 38) = 0.05, *p* = 0.95, *ղ2p* = 0.001, small). Both groups demonstrated a moderate effect of exercise on self-reported arousal (moderate intensity group: *d* = 0.65, vigorous intensity group: *d* = 61).

## 4. Discussion

The aim of the current study was to investigate the effect of a single bout of aerobic exercise conducted at moderate or vigorous intensity on motor practice, 24-h retention, and transfer. The findings indicated an overall improvement in golf putting accuracy, albeit with no statistical difference between the exercise groups across blocks of practice (see Figure 2). Similarly, no group differences emerged at 24-h retention or in a transfer test (putting with a shorter distance to the target). Both groups also increased their working memory performance as well as self-reported arousal after exercising, albeit with no significant differences between the moderate intensity group and vigorous intensity group (see Figure 3 and Figure 4).

In this study, the results indicated that conducting aerobic exercise at different intensities did not appear to introduce significant difference in the rate of improvement during the practice/acquisition phase of motor learning (see Figure 2). Similar results have been observed in other studies over the past decade. Conducting a bout of aerobic exercise before motor practice has consistently been found to not improve learning rates across blocks of practice compared to non-exercise (rest) conditions (see [27] and [29] for other patterns of results). This overall pattern of similarity in immediate motor skill improvement between exercising and non-exercising conditions is evident in studies on bouts of acute vigorous-to-heavy intensity cardiovascular exercise [6,31,32,55,56,57,58] as well as bouts of acute moderate intensity exercise [11,30,59]. The results of these studies, and those of ours, thus clearly indicate that acute aerobic exercise conducted at moderate-to-vigorous intensity levels for 20–30 min has no effect on subsequent acquisition/practice phase of the motor learning process.

At first sight, this null effect on initial motor practice after aerobic exercise appears somewhat contradictory when viewed in light of the cognitive–energetic models [14]. Under this perspective, moderate levels of exercise-induced arousal are expected to facilitate learning, and vigorous intensity should compromise learning [16,17]. This hypothesis is clearly not confirmed in the results of the current study or in most studies in the previous paragraph concerning motor learning. In studies that investigated the effects of acute aerobic exercise on various elementary cognitive measures, however, the non-linear relationship between exercise-induced arousal and task performance has been confirmed in a meta-analysis [19]. A distinction must thus be made between the lack of immediate effects of cardiovascular exercise on immediate motor practice compared to the positive immediate effects on measures, such as processing speed, reaction time, and sustained attention [60]. There are relatively few studies that have investigated the effects of acute exercise on learning cognitive skills, in which both positive [61] and no effect has been observed [62].

The null effect of elevated exercise-induced arousal on the practice phase of motor learning, as found in previous studies as well as ours, might be explained by the nature of the practicing motor skills. Although group-level data (as depicted in Figure 2) typically illustrates overall improvements in motor skill across blocks of practice, individual learning curves contain substantial trial-to-trial variability [63]. This introduces a certain degree of dispersion in group-level data, which is manifested as a high degree of overlap in between-group practice scores even without any elevated exercise-induced arousal. Thus, exercise effects that cannot be expected to be substantial given the results of meta-analysis [19] might not be detectable within the baseline levels of individual- and, consequently, group-level variability in motor practice scores. Although some studies have found exercise-induced differences in immediate motor practice [27,29], this can be explained by the type of motor task applied in various studies. As task complexity increases, i.e., tasks contain more degrees of freedom, the trial-to-trial variability is also expected to increase [64]. Thus, motor tasks with a relatively low range of movement (e.g., static force production) might indicate comparable exercise-induced effects to simple cognitive measures (e.g., reaction time), while motor tasks with a much higher demand for coordination, such as the putting task in the current study, do not show any acute effects of exercise due to high trial-to-trial variability. The generalizability of acute exercise effects upon motor practice clearly must be further examined in relation to a variety of motor skills.

It has been suggested that exercise interacts more with mechanisms underlying the consolidation of motor skills compared to the processes associated with the motor skill acquisition phase. In neuroendocrinological theory, this effect emerges through acute cardiovascular exercise triggering neuroplasticity in cortico-spinal pathways [9] and increased excitability of central brain areas for motor learning [10]. This, in turn, is related to moderate increases in the magnitude of brain neurochemicals, which might originate from the lactate released from skeletal muscles during exercise [7,8]. The theory postulates that moderate intensity exercise is favorable, given that a very high intensity might introduce substantial and task-compromising levels of neurochemicals. In the current study, however, no differences were found between exercising at 50% vs. 75% of HR_MAX_ (see Figure 2) on the 24-h retention of a golf putting skill. In previous studies, the evidence for exercise-induced improvement of motor retention appears mixed. Moderate intensity exercise has demonstrated both improved [59] and no significant effect [29,30] on motor retention. Similarly, vigorous intensity aerobic exercise has been found to improve short- and long-term retention [6,31,32,56], albeit other studies have not observed similar patterns of results [55,58]. The current base of studies thus does not indicate a systematic effect of acute exercise on the retention of motor skills, and a clear avenue for further research could disentangle the specific relationship between exercise intensity, type of motor skill, and short/long-term retention.

The role of acute exercise in promoting motor transfer has, by the authors’ knowledge, rarely been examined in previous studies. Improved exercise-induced transfer, referring to the influence of previous practice on performing a skill in a new context [65], might occur through similar neuroendocrinological mechanisms as proposed for motor retention. In the current study, however, no such improved transfer was observed (see right-hand side, Figure 2). Similarly, Neva et al. [59] did not find significantly increased exercise-induced transfer from right-hand practice towards left-hand (non-practiced) performance in a visuomotor reaching task. The relatively scarce amount of data thus indicates no exercise-induced enhancement of motor transfer.

As depicted in Figure 3, conducting aerobic exercise in the moderate-to-vigorous range improved post-exercise assessment of working memory. However, no difference was observed between the study groups, and the overall exercise-induced improvement amounted to a medium-level effect size. This appears to be in line with meta-analytical comparisons of effects by McMorris et al. [3]. In their study, potentially large gains in aspects of working memory were found after intermediate intensity exercise. Although our effect size was at a more moderate level compared to the overall effect sizes for the response times obtained by McMorris et al. [3], this might be explained by the application of a different working memory task. Included studies in the meta-analysis typically reported measures, such as reaction time, while our working memory task consisted of reporting number letter strings. Overall, it supports the hypothesis that aspects of working memory are selectively affected by increased exercise-induced arousal (see Figure 4), possibly associated with increases in the magnitude of brain catecholamines leading to faster processing, as predicted by neuroendocrinological theory [15,20].

There are limitations to this study that should motivate further studies. Participants with different exercise levels were not systematically recruited, and we did not measure participants’ physical capacity directly. These might be important independent variables in the examination of the interaction between exercise and learning, as indicated by previously published data [66]. In this study, however, participants in both groups were of a similar age and BMI, had similar resting heart rates, and reported comparable levels of physical activity/sedentary behaviors (see Table 1) at the group level. The study also consisted of a convenience sample including both men and women, albeit no statistical differences between males and females in motor learning were found. Furthermore, the study examined a relatively short-term retention (24 h) after motor practice. It has been suggested that motor learning (consolidation) is best studied at various intervals of delayed retention [67] However, previous acute exercise learning studies that have applied 24-h and 7-day retention intervals did not find differences between these timepoints [6,32,55,58]. In a previous study, we did not find any effect of aerobic exercise on 7-day retention after a 4-week practice period [68]. The potential for exercise-induced long-term effects on motor learning thus awaits further work.

## 5. Conclusions

In summary, the results of the current study suggest that acute aerobic exercise at moderate-to-vigorous intensity does not induce differences in the motor practice phase or at testing during 24-h retention and transfer. These exercise intensities also appear to induce similar effects on self-reported arousal and working memory. The postulated detrimental effects of vigorous exercise from cognitive–energetic and neuroendocrinological approaches were thus not found. This warrants some further work on possible moderators and the specific dose–response relationship between acute exercise, arousal, and motor learning processes.

## Figures and Tables

**Table 1:** Descriptive statistics across the two study groups. All values are mean (+/− SD) unless otherwise reported.

Variable		Moderate intensity (*n* = 22)	Vigorous intensity (*n* = 18)	*p* ^1^
Male/Female (*n*)		12/10	8/10	0.52 ^2^
Age (years)		23.77 (1.88)	23.83 (2.15)	0.93
BMI (weight/height^2^)		23.08 (2.42)	23.49 (2.88)	0.63
Letter Number Sequencing		9.73 (2.43)	10.50 (3.26)	0.39
Ravens progressive matrices		51.67 (3.73)	50.72 (3.29)	0.40
Resting heart rate (beats/min)		72.78 (10.89)	73.33 (8.48)	0.86
Leisure time PA (Hours/week)	Walking	2.34 (2.32)	1.75 (1.33)	0.36
	Moderate	1.94 (3.10)	1.63 (1.96)	0.72
	Vigorous	2.25 (1.86)	2.91 (2.78)	0.40
Total sedentary hours/week	Weekdays	42.74 (16.32)	44.09 (21.62)	0.82
	Weekend	13.64 (5.48)	14.64 (6.01)	0.61

PA Physical activity/exercise; BMI Body Mass Index; ^1^ Two-way ANOVA, ^2^ Pearson Chi-Square for proportion of males/females across groups.
